# The relationship between scleral staphyloma and choroidal thinning in highly myopic eyes: The Beijing Eye Study

**DOI:** 10.1038/s41598-017-10660-z

**Published:** 2017-08-29

**Authors:** Ling Xiao Zhou, Lei Shao, Liang Xu, Wen Bin Wei, Ya Xing Wang, Qi Sheng You

**Affiliations:** 1 Beijing Tongren Eye Center, Beijing Tongren Hospital, Capital Medical University, Beijing, China; 2 Department of Ophthalmology, The First Affiliated Hospital of Xi’an Medical University, Xi’an, Shaanxi China; 3 Beijing Institute of Ophthalmology, Beijing Tongren Hospital, Capital Medical University, Beijing, China

## Abstract

Based on the Beijing Eye Study 2011, a detailed ophthalmic examination was performed including spectral-domain optical coherence tomography (SD-OCT) with enhanced depth imaging for measurement of subfoveal choroidal thickness (SFCT) and relative height of posterior scleral staphyloma. OCT images were obtained in 103 highly myopic eyes (≤−6.00 diopters) and 227 normal eyes. The mean SFCT in highly myopic eyes was 110.6 ± 85.2 μm (range, 3 to 395 μm). The SFCT of high myopia without posterior scleral staphyloma(55 eyes) was 157.79 ± 85.18 μm, which was significantly greater than that (54.94 ± 49.96 μm) of high myopia with posterior scleral staphyloma (48 eyes) (P < 0.001). In multivariate analysis, posterior scleral staphyloma was the most important factor of choroidal thinning in high myopia (F = 22.63; P < 0.001), then age (F = 19.14; P < 0.001), axial length (F = 17.37; P < 0.001) and gender (F = 17.31; P < 0.001). The SFCT in highly myopic eyes is very thin and undergoes further thinning with increasing age and axial length (refractive error). Posterior staphyloma formation was a key factor in choroidal thinning in highly myopic eyes and to be a good indicator for risk management of choroidal thinning. Abnormalities of the choroid may play a role in the pathogenesis of myopic degeneration.

## Introduction

In Asia, Europe, and some races in the United States, a major cause of visual impairment is high myopia^1–3^. Generally, high myopia is related to axial extension of the eyeball, which can cause a variety of abnormalities in fundus, such as lacquer cracks in the Bruch membrane, posterior staphyloma, choroidal neovascularization (CNV), and chorioretinal atrophy^4–6^. Based on these findings, we defined the pathologic myopia as myopic refractive error equal or more than 6 diopters (D); an axial length more than 26.5 mm; or posterior pole abnormalities with high refractive errors^7–9^. Histologic studies suggested that choroid was thinner in high myopia, for choriocapillaris lost in some areas^10^. The choroid circulation receives about 95% of blood from the ophthalmic artery, and provides oxygen and nutrition to the outer retinal layers. Therefore, the choroidal circulation may play an important role in the retinal dysfunction and vision loss. Posterior staphyloma formation is significantly associated with macular hole, retinal detachment and myopic foveoschisis, so it is recognized as a hallmark of high myopia.

Enhanced depth imaging spectral-domain optical coherence tomography (EDI SD-OCT) has supported a way to detect choroidal thickness *in vivo* ^11^. Using this method, several studies have reported the SFCT in normal subjects and patients with abnormalities in fundus^12–14^, including high myopia^15, 16^. However, these studies were hospital-based studies which probably may not avoid a selection bias. Therefore, based on a population-based study, we attempted to study the SFCT in highly myopic eyes with EDI SD-OCT and to identify the risk factors for development of choroidal thinning with high myopia.

## Method

### Inclusion criteria

The Beijing Eye Study is a population-based prospective cohort study with an age of 50 + years in Northern China. The study has been described in detail^17, 18^. According to the Declaration of Helsinki, The Medical Ethics Committee of the Beijing Tongren Hospital had approved the study protocol and all participants had given informed consent. The Beijing Eye Study included 4439 subjects out of 5324 subjects invited to participate, corresponding to a response rate of 83.4% at the first time of the study in 2001. Participants were invited for the second five-year follow-up examination in 2011, at which time the SD-OCT was first performed on the participants. Of the 4403 subjects who agreed to participate at baseline, 3468[1963 (56.6%) women] agreed to continue to participate in 2011, corresponding to a response rate of 78.8%. The mean age of all participants was 64.6 ± 9.8 years (median, 64 years; range, 50–93 years).

Based on the data of Beijing Eye Study 2011, we conducted a cross-sectional study of highly myopic patients (spherical equivalent refractive error at least -6 D). Out of the total 3468 subjects, 135 (1.95%) eyes [prevalence rate (mean ± SE): 1.95 ± 0.17%; 95% CI: 1.62%, 2.27%] of 93 (2.68%) subjects (prevalence rate: 2.68 ± 0.27%; 95% CI: 2.14%, 3.22%) were regarded as highly myopic eyes. 103 eyes of 78 (83.9%) participants (50 (64.1%) women) have sufficient quality OCT images for examination. For 15 (16.1%) people, OCT images were not taken or images could not be assessed owing to the serious opacity of refractive media. Compared with the highly myopic subjects with OCT images, the group without images was significantly older (71.3 ± 7.7 years versus 64.8 ± 8.5 years; P = 0.007; 95% CI: 1.87, 11.29) but did not vary significantly in gender (P = 0.85) and refractive error (P = 0.67).

As control, 227 apparently normal eyes of 227 people were randomly selected from the base, with age and gender matched in proportion to the population. The exclusion criteria included amblyopia, glaucoma, uveitis, diabetic retinopathy, retinal vascular abnormalities, laser treatment, drusen, severe chorioretinal atrophy, macular scarring, refractive surgery, intraocular surgery (such as cataract extraction and vitrectomy), which were found by fundus photographs and questionnaire. Besides, patients with severe cataract, moderate to severe chorioretinal atrophy and pathologic structures were excluded resulting from SD-OCT.

### Basic examination

**Figure 1:**
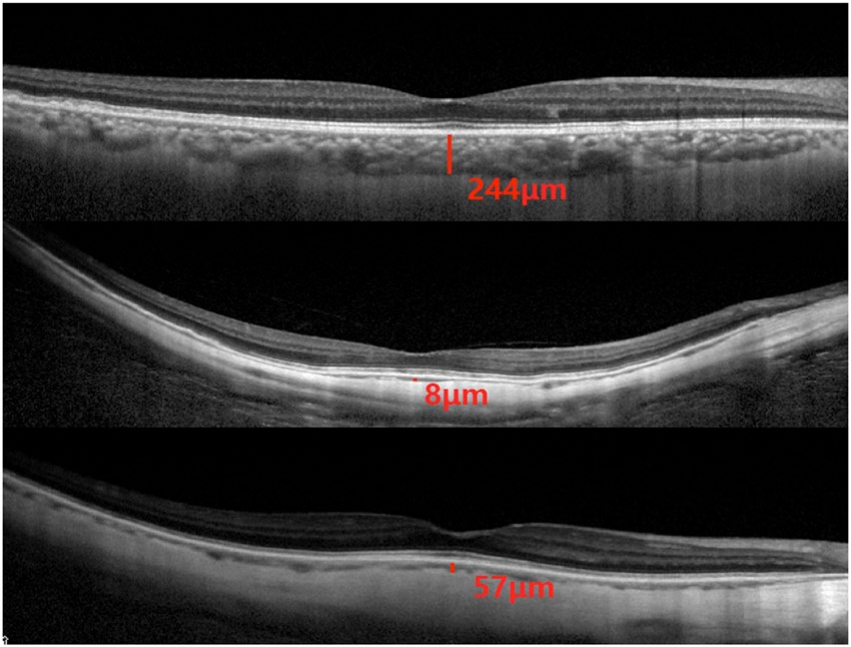
Cross-sectional imaging of the choroid using enhanced depth imaging optical coherence tomography (OCT). Subfoveal choroidal thickness was defined as the vertical distance from the hyperreflective line of the Bruch’s membrane to the hyperreflective line of the inner surface of the sclera. OCT measured (top line) 244 μm in a normal eye of a 55-year-old female, and (second line) 8 μm in an eye with -19.00 diopters (D) of a 70-year-old female, and (bottom line) 57 μm in an eye with -9.50D of 70-year-old male.

**Figure 2:**
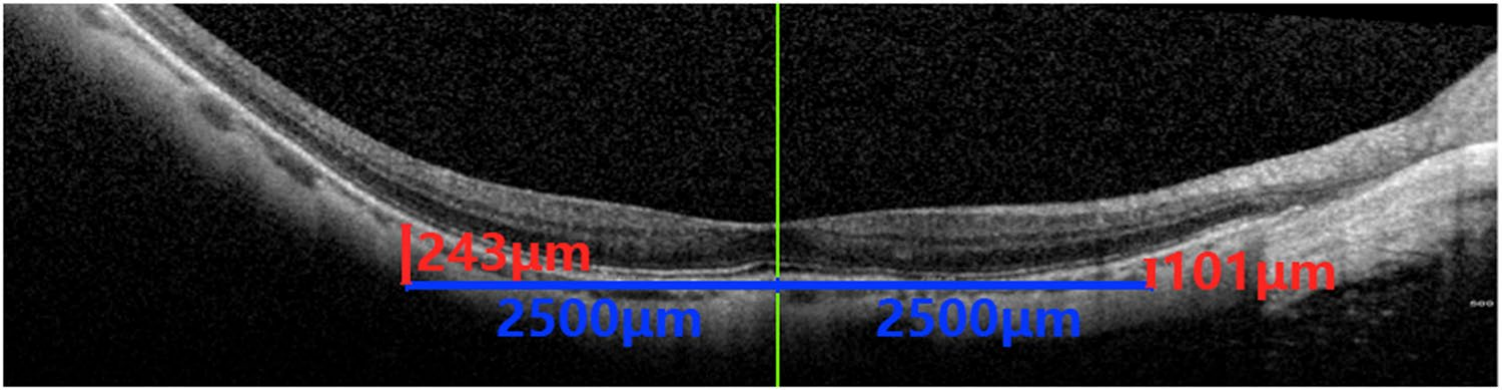
Vertical line was made 2.5 mm away from macular fovea to intersect with the horizontal line, and the vertical distance was measured as the relative height of scleral staphyloma.

After getting an informed consent, all study participants underwent an interview with standardized questions about their family and personal information. Then they went for fasting blood test of blood lipids, glucose and glycosylated hemoglobin HbA1c. Blood pressure, body height and weight and the circumference of the waist and hip were recorded. A detail ophthalmic examination was preformed. We measured the subfoveal choroidal thickness (SFCT) using a spectral domain optical coherence tomography (SD-OCT; Spectralis®, Wavelength: 870 nm; Heidelberg Engineering Co., Heidelberg, Germany) with enhanced depth imaging (EDI) modality. SFCT was the vertical distance from the hyperreflective line of the Bruch’s membrane to the hyperreflective line of the inner surface of the sclera (Fig. 1). Seven sections, each comprising 100 averaged scans, were obtained in an angle of a 5 to 30 degree rectangle centered onto the fovea. The horizontal section running through the center of the fovea was selected for further analysis. Detailed measuring method can be seen in the references^19, 20^. The measurements were performed by two examiners (K.F.D, L.S) independently and the interobserver reproducibility was proved high (r = 0.992, P = 0.000). The relative heights of scleral staphyloma at the posterior pole were measured, and the method is as follows: 31 sections were scanned to cover 8 mm * 8mm areas of macular fovea, which means almost all of the posterior pole was covered, and each section was the average image of 20 times scanning images. Vertical line was made 2.5 mm away from macular fovea to intersect with the horizontal line, and the vertical distance was measured as the relative height of scleral staphyloma. The relative heights of scleral staphyloma was measured in nasal, temporal, superior and inferior quadrants, and the four points 2.5 mm away from the fovea of every quadrants were measured (Fig. 2). If the point in corresponding quadrant was anterior to the horizontal line at the fovea, we recorded it as a positive value, and the opposite case was recorded as a negative value. All measurements were performed using the Heidelberg Eye Explorer software (version 5.3.3.0; Heidelberg Engineering Co., Heidelberg, Germany).

### Statistical analysis

Statistical analysis was performed by using a commercially available statistical software package (SPSS for Windows, version 17.0, SPSS, Chicago, IL, USA). SFCT was described by the mean values (presented as mean ± SD). Categorical variables were assessed individually with the chi-square test, and when the expectancy of less than 5, the Fisher’s exact test was used. Continued data were analyzed using an independent sampled t-test. Simple linear regression was calculated for variations in SFCT relative to systemic and ocular risk factors in highly myopic eyes. Multiple linear regression was used to evaluate the explanatory variables with regard to the dependent variable. Only the right eye of the participant was assessed in linear regression analysis. 95% Confidence intervals (CI) were presented. All P-values were 2-sided and were considered statistically significant when < 0.05.

## Result

Highly myopic group contained 103 eyes of 78 patients and control group contained 227 eyes of 227 participates. Table 1 showed the results of comparison of demographic and ocular characteristics between highly myopic group and control group, included age, refractive error and SFCT, *et al*.

**Table 1:** Comparison of demographic and ocular characteristics between highly myopic and normal eyes.

Factors	Mean ± SD		P (Two-tailed)
	Highly myopic eye	Normal eye	
Age	64.5 ± 8.3	65.2 ± 8.8	0.471
Gender (male/female)	37/66	89/138	0.569*
Height (cm)	162.2 ± 9.5	161.2 ± 7.9	0.421
Weight (kg)	64.2 ± 12.7	66.2 ± 11.2	0.206
Best-corrected visual acuity	0.67 ± 0.29	1.04 ± 0.08	<0.001
Refractive error (D)	−9.54 ± 3.57	0.35 ± 0.67	<0.001
Axial length(mm)	26.62 ± 1.98	23.07 ± 0.72	<0.001
IOP (mm Hg)	15.2 ± 2.5	14.5 ± 2.8	0.051
Subfoveal retinal thickness (μm)	208.9 ± 54.4	216.8 ± 17.5	0.149
Subfoveal choroidal thickness (μm)	110.6 ± 85.2	263.0 ± 92.9	<0.001

^*^The gender assessed individually with the chi-square test, other factors assessed with independent-sample t test.

Stepwise multiple linear regression analysis confirmed that posterior scleral staphyloma was the most important factor of SFCT in high myopia (F = 22.63; P < 0.001), then age (F = 19.14; P < 0.001), axial length (F = 17.37; P < 0.001) and gender (F = 17.31; P < 0.001). Table 2 showed the results of multiple regression analysis. There were 48 eyes with high myopia that had staphyloma and 55 eyes without staphyloma, which OCT images were available. Comparing with the highly myopic eyes without staphyloma (157.79 ± 85.18), the mean SFCT was significantly decreased in the high myopia with staphyloma group (54.94 ± 49.96 μm) (P < 0.001). The relationship of progress of central choroidal thinning with posterior scleral staphyloma was analyzed. Demography and eye data of the above two groups were shown in Table 3. The linear regression model showed that age was associated with SFCT in both groups with or without posterior scleral staphyloma (b = −3.71, P = 0.001; b = −4.32, P = 0.003, respectively). Axial length was related to the SFCT in the posterior scleral staphyloma group (b = −11.82, P = 0.011). But in the group without posterior scleral staphyloma, they had no significant correlation (P = 0.284). Gender was not related to the SFCT in both groups (P = 0.286, P = 0.726, respectively).

**Table 2:** Multivariate analysis of associations between SFCT in highly myopic eyes (as measured by enhanced depth imaging of spectral domain optical coherence tomography) and ocular and general parameters in the Beijing eye Study 2011.

Parameter	Unstandardized Coefficients(B)	95% Confidence Interval	Standardized Coefficients(beta)	P-Value
Staphyloma	−45.35	−90.81, 0.11	−0.26	0.050
Age (years)	−5.12	−7.39, −2.85	−0.48	<0.001
Axial Length (mm)	−19.75	−30.70, −8.81	−0.44	0.001
Gender	−60.32	−104.75, −15.89	−0.31	0.009

R^2^ = 0.63.

**Table 3:** Comparison of demographic and ocular characteristics between highly myopia with and without staphyloma.

Factors	Mean ± SD		P (Two-tailed)
	Staphyloma group	No staphyloma group	
Age	66.6 ± 8.2	63.8 ± 9.2	0.182
Gender (male/female)	7/25	19/19	0.015*
Height (cm)	158.4 ± 8.7	165.6 ± 9.5	0.001
Weight (kg)	60.3 ± 11.5	67.3 ± 13.1	0.017
Best-corrected visual acuity	0.56 ± 0.30	0.75 ± 0.26	0.006
Refractive error (D)	−11.61 ± 4.05	−7.48 ± 1.93	<0.001
Axial length(mm)	27.75 ± 1.77	25.70 ± 1.65	<0.001
IOP (mm Hg)	14.6 ± 2.1	15.6 ± 3.2	0.136
Subfoveal retinal thickness (μm)	188.3 ± 53.5	214.9 ± 27.6	0.015
Subfoveal choroidal thickness (μm)	54.9 ± 50.0	157.8 ± 85.2	<0.001

^*^The gender assessed individually with the chi-square test, other factors assessed with independent-sample t test.

In patients with high myopia, the relative heights of posterior scleral staphyloma 250 μm away from the macular foveola in four quadrants were measured. Analysis of variance and intergroup comparison showed that the average height of the superior (277.8 ± 232.3 μm) and temporal (246.6 ± 156.8 μm) posterior scleral staphyloma was larger (P = 0.52), followed by the nasal (165.4 ± 154.4 μm, P = 0.002) and the inferior quadrant (99.4 ± 209.7 μm, P < 0.001) (Table 4). Univariate regression analysis showed the relative height of nasal posterior scleral staphyloma (250 μm from the macular fovea) was negatively correlated with SFCT (b = −0.44,P = 0.01,95% CI: −2.39, −0.31), nasal choroidal thickness (250 μm from the macular fovea) (b = −0.35, P = 0.05, 95% CI: −2.81, 0.00) and diopter (b = −0.52, P = 0.02, 95% CI:−34.07, −8.37), and marginally positively correlated with axial length (b = 0.35, P = 0.07). But it had no significant correlation with age (P = 0.75), gender (P = 0.75) and best corrected visual acuity (BCVA) (P = 0.34). Multivariate regression analysis showed the relative height of nasal posterior scleral staphyloma was still significantly negatively correlated with diopter (b = −0.37, P = 0.04, 95% CI: − 29.1, −0.42) and with SFCT (b = −0.37, P = 0.04, 95% CI: −0.261, −0.03). But it had no significant correlation with the nasal choroidal thickness (P = 0.22) and axial length (P = 0.51). However, univariate analysis showed the relative height of temporal (P = 0.08), superior (P = 0.79) and inferior (P = 0.86) posterior scleral staphyloma had no significant correlation with SFCT as well as choroidal thickness of corresponding quadrants (all P > 0.1).

**Table 4:** Relative heights of posterior scleral staphyloma 250 μm away from the macular foveola.

Quardrant	Height (μm)	Intervals (μm)	Analysis of variance P values
	Average ± standard deviation	(minimum, maximum)	
Superior	277.8 ± 232.3	(−266, 833)	<0.001
Temporal	246.6 ± 156.8	(12, 710)	
Nasal	165.4 ± 154.4	(−85, 646)	
Inferior	99.4 ± 209.7	(−441, 560)	

## Discussion

In our population-based study, the results of the mean SFCT of high myopia as measured in our study were somewhat different from those reported previously. In Fujiwara T’s study on 55 highly myopic eyes with a mean age of 59.7 years, SFCT was 93.2 μm, which was thinner than our results^15^. But when we took the age difference between both study populations into account, the SFCT measurements in our study were even slightly lower than the results of previous report. Correspondingly, Ikono and coworkers measured the SFCT in 31 highly myopic Japanese with a mean age of 51.7 years and found a mean value of 100.5 μm^16^, which was three times thinner than our result after correction for age. Reasons for differences between various studies in the SFCT measurements of high myopia could be differences in the myopic refractive error of the study populations and, potentially, in the anatomy of the globes with ethnic differences.

This study found that posterior scleral staphyloma was the most important factors affecting choroidal thickness of high myopia (F = 22.63, P < 0.001) by using multivariate regression analysis. Then, the statistical comparison of the systemic and ocular features of highly myopia with and without staphyloma showed that the group without staphyloma had obviously better BCVA, less myopia, shorter axial length, thicker subfoveal retina, and even taller and fatter than those in group with staphyloma. For the SFCT, the group without staphyloma(157.79 ± 85.18) was nearly 2 times more than staphyloma group (54.94 ± 49.96 μm) (P < 0.001). Therefore, the posterior scleral staphyloma can serve as a hallmark of high myopia. Previous researches pointed out that the formation of the posterior scleral staphyloma was closely related to the occurrence of some posterior retinopathy, including macular hole, retinal detachment and myopic retinoschisis^21^. It may originate from inward retinal traction by huge posterior scleral staphyloma to cause neuroretinal detachment or tear. And to a certain extent, this process may be involved in the formation of vitreous folds, macular pucker and the damage of inner limiting membrane^22^. Based on the study of ultrasonic diagnosis, posterior scleral staphyloma was close to specific fundus lesions of high myopia, such as pathological changes of the vitreous body, lacquer cracks, pigment epithelial damage and choroidal atrophy^23^. Posterior staphyloma height was proved to have significant correlation with axial length and refractive error^16^. However, the axial length can be as low as 24.5 mm in pathologic myopia eyes, while it can be as long as 27.3 mm in emmetropia eyes^9^. In addition, the relationship between threshold of the axial length and myopic maculopathy is still unknown. Globe expansion may induce disproportional choroidal thinning, especially at the central fovea. In the recent study, staphyloma was the top factor related to SFCT, instead of age and axial length (Table 2). Thus, posterior staphyloma height appears to bea good indicator for risk management of choroidal thinning.

In this study, the relative heights of posterior scleral staphyloma 250 μm from the macular foveola in four quadrants of patients with high myopia were further measured. Final results showed that the average height of superior and temporal posterior scleral staphyloma was bigger, followed by the nasal and the lowest was inferior. The reason for the obvious differences of the relative height of posterior scleral staphyloma among different quadrants is unclear, which may relate to axial elongation, vitreous traction as well as the effect and the direction of gravity. In addition, this study found that the distribution law of the relative height of posterior scleral staphyloma was the same with that of the height of incomplete posterior vitreous detachment in each quadrant. Another study of our research team showed that, for incomplete posterior vitreous detachment, the highest quadrant was superior and temporal, followed by the nasal and the lowest was inferior^19^. Thus, we concluded that the formation and severity of posterior scleral staphyloma might be affected by forces generated by axial elongation and vitreous liquefaction and detachment in highly myopic eyes.

Univariate regression analysis of the relative heights of posterior scleral staphyloma revealed that the relative height of nasal posterior scleral staphyloma negatively correlated with SFCT, nasal choroidal thickness and diopter, and it marginally, positively correlated with the axial length. Further multivariate regression analysis found that the relative height of nasal posterior scleral staphyloma still significantly negatively correlated with diopter and SFCT, but it had no significant correlation with the nasal choroidal thickness and axial length. The results showed that the degree of posterior scleral staphyloma, especially posterior scleral staphyloma in macular nasal quadrant may be the most important factors for macular choroidal thinning in high myopia. However, univariate regression analysis indicated that the relative height of temporal, superior and inferior posterior scleral staphyloma had no significant correlation with SFCT and choroidal thickness of corresponding quadrants. Only the reason for the significantly negative correlation of the relative height of nasal posterior scleral staphyloma with SFCT is still uncertain. Possible explanation for this was that area of 250 μm away from macular nasal side (the measuring site of the relative height of posterior scleral staphyloma) lies between the fovea and the optic disc and the two regions are closely adhesive with vitreous body^19^. When nasal posterior scleral staphyloma in high myopia forms, the traction of the choroid and bilateral retina in the areas of macular fovea and optic disc concentrates and the resultant force is relatively big to subsequently cause abnormal choroidal structure and function and even atrophy due to its twisting. When posterior scleral staphyloma in other quadrants formed, the impact of nasal posterior scleral staphyloma on SFCT does not appear due to the absence of close vitreous adhesion to the optic disc, which causes relatively small disturbance to the corresponding choroidal areas.

Univariate analysis of this study indicated that the relative height of nasal posterior scleral staphyloma had marginally positive correlation with axial length, but multivariate analysis showed it had no significant correlation with axial length. Therefore, axial length may be a confounding factor. Another study suggested that the depth of posterior scleral staphyloma associated with axial length and refractive error^24^. However, the axial length of patients with pathological myopia was also in the normal range, for example 24.5 mm, and the axial length of emmetropia can increase to 27.3 mm^9^. The threshold of axial length causing myopic maculopathy is still unclear. Eye elongation may induce uneven choroidal thinning, which is thinner in the foveal area with lower perfusion. We proposed that in place of age and axial length, the posterior scleral staphyloma may become to the most important factor of subfoveal choroidal thickness in high myopia (Table 2). Then, the depth of the posterior scleral staphyloma, especially the relative height of nasal posterior scleral staphyloma may be used as an important predictor of the risk of choroidal atrophy in high myopia. However, the causal association between posterior scleral staphyloma and choroidal thinning is still unclear, the correlation with specific fundus lesions in myopia remains to be further investigated. In some cases, the scleral staphyloma may be far away from the fovea, and OCT can not recognize them very well, so the three-dimensional magnetic resonance imaging would be taken to evaluate the scleral staphyloma and to complement the conclusion in the future study.

In conclusion, SFCT in highly myopic eyes (110.6 ± 85.2 μm) was markedly thinner than normal eyes (263.0 ± 92.9 μm). It was most associated with posterior staphyloma, followed by age, axial length and gender. These observations may reveal that posterior staphyloma was an independent factor of choroidal thinning in highly myopic eyes, and abnormalities of the choroid may play a role in the pathogenesis of myopic degeneration.
